# Retinopathy of prematurity in Kenya: prevalence and risk factors in a hospital with advanced neonatal care

**DOI:** 10.11604/pamj.2018.29.152.14046

**Published:** 2018-03-15

**Authors:** Oscar Onyango, Sarah Sitati, Lucia Amolo, Florence Murila, Susan Wariua, Gacheri Nyamu, Moses Lango, Atul Patel

**Affiliations:** 1 Nairobi Hospital, Department of Ophthalmology, Nairobi, Kenya; 2 Sabatia Eye Hospital, Department of Pediatric Ophthalmology, Wodanga, Kenya; 3 Nairobi Hospital, Department of Neonatology, Nairobi, Kenya

**Keywords:** Retina, retinopathy, child eye health, prematurity, neonatal health, premature, retinopathy of prematurity

## Abstract

**Introduction:**

Increased survival of preterm babies in sub-saharan Africa has held to an increasing prevalence of Retinopathy of prematurity (ROP). This study was done to determine the ROP prevalence in a hospital with advanced neonatal care in urban Kenya.

**Methods:**

A hospital-based retrospective review of the records of premature infants screened for ROP between January 2010 and December 2015. Records of all premature infants screened for ROP in the neonatal unit and outpatient eye clinic were extracted. Information on Birth weights, Gestational age, Maternal risk factors (mode of delivery, pre-eclampsia/eclampsia) and Neonatal risk factors (neonatal sepsis, days on oxygen, blood transfusion) was recorded in a questionnaire then analysed.

**Results:**

103 infants were included in the study. Mean gestational age was 29.9 ± 2.2 weeks and the mean birth weight was 1280.1 ± 333.0 grams. Forty-three infants were diagnosed with ROP, a prevalence of 41.7%. Majority of these had Stage 1 or 2 ROP in Zone II, which spontaneously regressed with follow up. Nine infants were diagnosed with vision-threatening ROP (any Zone I disease or Stage 2/3 disease in Zone II with plus disease), a prevalence of 20.9%. All of these underwent laser treatment in the neonatal unit. The most significant risk factor was low gestational age. Other risk factors identified were: low birth weight and blood transfusions.

**Conclusion:**

ROP prevalence in sub-saharan Africa will match those in middle-income and high income countries in neonatal units with advanced care and low mortality.

## Introduction

About 60% of the world's preterm births occur in sub-Saharan Africa and Asia. In Kenya, pre-term births are estimated at 12.3% [1]. With improved neonatal systems in developing countries, survival rates for premature infants have increased [2, 3]. Many of these infants survive with disabilities, including visual complications. Retinopathy of prematurity (ROP) is now emerging as an important cause of ocular morbidity in low and middle income countries [4, 5]. Despite this, little is known about ROP in sub-Saharan Africa, Kenya included [2]. Lack of information has hampered the development of screening programs that would aid early detection and treatment of ROP. ROP screening and treatment has been ongoing in Nairobi Hospital since 2010. Survival rates for preterm infants is high and ROP has been identified as an upcoming epidemic in this hospital This study was done to determine the prevalence and risk factors associated with ROP in a hospital with advanced neonatal care in a developing country.

## Methods

A hospital-based retrospective review of the records of premature infants screened for ROP between January 2010 and May 2015.The Nairobi Hospital is a modern private hospital with advanced neonatal care, located in Kenya's capital of Nairobi. Neonatal care in this institution is comparable to that in many middle and high income countries. The hospital has a well-equipped neonatal unit, including a neonatal intensive care unit (NICU). There are five neonatologists, several resident pediatricians and trained neonatal unit nurses working in the unit. Ethical approval was obtained from the Nairobi Hospital Ethical Review Committee. A consent waiver was requested and approved prior to examination of patient records. Records of all premature infants born within the study period and screened for ROP in the neonatal unit and outpatient eye clinic were extracted. Information on Birth weights, Gestational age, Maternal risk factors (mode of delivery, pre-eclampsia/eclampsia) and Neonatal risk factors (neonatal sepsis, days on oxygen, mechanical ventilation, blood transfusion) was recorded in a questionnaire then analysed. The hospital has an in-house screening program and all babies born < 32 weeks gestational age or weighing ≤1500 grams are referred to the ophthalmologist for review. High risk infants who fall outside the screening criteria are also referred, for example, those with prolonged hospital stays due to mechanical ventilation, neonatal sepsis and surgical cases. A tracking system in the unit ensures that appointment reminders are given by the neonatologist/ pediatrician during clinic visits. Infants included in the study had been examined by a single vitreo-retinal specialist starting at 28 days of life and followed up one-two weekly until achievement of full retinal vascularisation or achieving 50 weeks post-natal age. Those with Type 1 ROP (any Zone I disease, Stage 2-3 disease in zone II with plus disease) received laser treatment within the neonatal unit within 48 hours of diagnosis. Those with Type 2 ROP (Stage 1-2 without plus in zone I, Stage 2-3 without plus in zone II) were followed up two weekly until regression of ROP or until vascularisation in zone III was achieved. On achieving zone III vascularisation, infants were then referred to the pediatric ophthalmologist for follow up.

## Results

71,888 infants were admitted to the Nairobi hospital neonatal unit during the study period. Of these, 148 were classified as premature (gestational age <37 weeks). 103 had been screened for ROP in the neonatal unit and outpatient eye clinic and the records of these infants were all retrieved. The male to female ratio was 1:1.1, with 54 (52%) males and 49 (47%) females. Mean gestational age was 29.9 ± 2.2 weeks and the mean birth weight was 1280.1 ± 333.0 grams. Table 1 shows a summary of the socio-demographic characteristics of included infants. Forty-three infants were diagnosed with ROP, a prevalence of 41.7%. Table 2 gives a summary of the ROP descriptions among the premature infants and their progression through subsequent follow up visits. Majority of these had Stage 1 or 2 ROP in Zone II, which regressed with follow up. The mean birth weight in this group was 1207.4 grams and mean gestational age 29.1 weeks. 17/43 (39.5%) received blood transfusions while in the neonatal unit, and 10/43 (23.3%) were diagnosed with neonatal sepsis. 33/43 (76.7%) were on mechanical ventilation. The main maternal risk factor identified was eclampsia/pre-eclampsia, with 12/43 (27.9%) infants having this risk. Nine infants were diagnosed with vision-threatening ROP (any Zone I disease or Stage 2/3 disease in Zone II with plus disease), a prevalence of 20.9%. All of these underwent laser treatment in the neonatal unit. Table 3 shows the socio-demographic data and risks identified in those with vision-threatening ROP. Finally, Table 4 shows the risk factors associated with development of ROP in this group of premature infants and their significance (p < 0.05). The strongest risk factor for development of ROP was gestational age, followed by mechanical ventilation. Other significant risk factors included low birth weight and prescence of blood transfusion. Although neonatal sepsis was present in more than half of the infants who developed ROP requiring treatment (5/9; 55.6%), it did not have statististical significance when the larger group was examined.

**Table 1:** Socio-demographic characteristics of premature infants, n=103

Variable	Frequency (%)
Male Female	54 (52.4) 49 (47.6)
Mean Gestational Age at birth (Weeks) Range	29.9 ± 2.2 25-37
Mean Birth Weight (Grams) Range	1280.1 ±333.0 650-1890
Mean Maternal age (Years) Range	33.3 ± 5.8 23-50
Mode of delivery C/S SVD	79 (76.7) 24 (23.3)
Mean post-natal age (PNA) at first exam (Days) Range	34.3 (2.2) 30-42

**Table 2:** ROP Descriptions, n=86 eyes

Variable	RE			LE		
	InitialExam(n=43)	Followup 1(n=14)	Followup 2(n=3)	InitialExam(n=41)	Followup 1 (n=8)	Followup 2(n=1)
Stage						
1	30	7	1	23	2	0
2	12	6	1	13	6	1
3	1	1	1	2	0	0
Zone						
1	2	0	0	0	0	0
2	16	9	0	15	1	0
3	20	3	2	17	6	1
Plus disease						
Mild	10	4	1	8	3	0
Moderate	3	1	0	1	0	0
Marked Plus disease	2	1	1	3	1	0
No plus disease	28	0	0	31	10	0

**Table 3:** Descriptions of infants with ROP requiring treatment, n = 9

Variable	Frequency (%)
Mean Birth Weight (grams)	29.2 ±1.8
Range	26.0-32.0
Mean Gestational Age (weeks)	1184 ±271.0
Range	870-1660
Days on mechanical ventilation	7 ±77.8
Median (IQR)	3 (1-10)
Baby with neonatal sepsis	
Yes	5 (55.6)
No	4 (44.4)
Received blood transfusion	
Yes	5 (55.6)
No	4 (44.4)
Mother diagnosed with eclampsia/pre-eclampsia	
Yes	3 (33.3)
No	6 (66.7)

**Table 4:** Risk factors among infants with ROP (n=103)

	ROP diagnosed		P value(p<0.05)
	Yes (%)	No (%)
**Mean gestation age at birth** (SD)	29.1 (1.9)	30.6 (2.2)	< 0.001
Range	25.0 - 33.0	26.0 - 37.0	
**Mean birth weight** (SD)	1207.4 (254.9)	1334.4 (301.5)	0.027
Range	725.0 - 1800.0	650.0 - 1890.0	
**Documented sepsis**			
Yes	10 (25.0)	10 (17.9)	0.396
No	30 (75.0)	46 (82.1)	
**Baby got blood transfusion**			
Yes	17 (40.5)	11 (19.0)	0.018
No	25 (59.5)	47 (81.0)	
**Blood-culture positive sepsis**			
Yes	3 (8.1)	1 (1.9)	0.302
No	34 (91.9)	52 (98.1)	
**Baby required mechanical ventilation**			
Yes	33 (78.6)	29 (50.0)	0.004
No	9 (21.4)	29 (50.0)	

## Discussion

The question of whether or not ROP is an upcoming epidemic in Africa has been under debate for some years now. Data on ROP in sub-Saharan Africa is scanty and studies in the schools for the visually impaired and hospitals in easetern Africa show low prevalence of retinal diseases, ROP included [6-8]. It is thought that high neonatal mortality in low income countries, especially among prematures, has contributed to this trend [2]. Neonatal systems in Africa have improved in the recent past, renewing the question of ROP prevalence. The above study shows that ROP prevalence will match those in middle and high income countries in hospitals with advanced neonatal care. The prevalence of ROP in this study was 41.7%. Adio et al in Nigeria demonstrated a similar picture, with an ROP prevalence of 47.2% [9]. This prevalence matches those in middle-income countries where ROP has been identified as a new epidemic [10, 11]. It is however higher than that noted in a previous Kenyan study done in the same city. Wanjala et al, in a study done in two government hospitals in Kenya, had an ROP prevalence of 16.8% [12]. It is not uncommon to find varying ROP prevalences within the same country, depending on the level of neonatal care present [2]. Variation in infant mortality could also account for these differences. The infant mortality recorded during this study was 4.6% versus that recorded by Wanjala et al at 24.5%. Studies have also shown that infant mortality is skewed towards lower gestational age and birth weight [13], thus the cohort with the highest likelihood of developing ROP is also the one that is least likely to survive. This is especially true of sub-saharan African countries, where neonatal services are poorly developed. Low gestational age and low birth weight have been consistently identified as important risk factors for development of ROP [14-18]. Our study therefore shows that ROP prevalence is directly proportional to neonatal mortality and that improved neonatal systems may change picture of ROP blindness with time within the same setting [19, 20]. In the ROP descriptions, majority of the infants were found to have Type 2 ROP (non-vision threatening), most of which regressed spontaneously with follow up. Nine infants (9/103 (9.3%)) were diagnosed with Type 1 (vision threatening) ROP and underwent laser treatment in the neonatal unit by a retina specialist. Prevalences of vision-threatening ROP vary in different countries and with different races. Black race has also been thought to be a protective factor in development of vision-threatening ROP [21, 22]. This theory was supported by a Nigerian study where, despite a high ROP prevalence of 47.2%, none of the infants developed ROP requiring treatment [9]. The picture shown in our study was different, with vision threatening ROP matching that seen in middle income and high income countries with good neonatal care [23-25]. We propose the theory that black infants are just as likely to develop vision threatening ROP as those of other races. However, all the children in this study were of black African race, thus it was not possible to make a significant race comparison. When examining the risk factors associated with vision-threatening ROP, univariate analysis showed that gestational age was the most predominant risk factor followed by mechanical ventilation, low birth weight and blood transfusions. Several risk factors have been consistently identified for development of ROP, and those in this study matched with other studies [15, 23, 26, 27]. Although sepsis was not a significant risk factor in our study, other African studies have identified it as an important factor in the development of ROP [9,12]. This study had a limitation of being a retrospective study. Despite having no missing records, a prospective study would have been more useful in determining the ROP prevalence.

## Conclusion

In conclusion, the Nairobi hospital study shows that ROP prevalence in Africa will match that in other countries as neonatal care continues to improve. While hospitals with poor neonatal care may continue to see scanty ROP cases, hospitals with advanced neonatal care must develop rigorous screening programs so that no case of ROP is missed.

### What is known about this topic

- Preterm births are high in sub- Saharan africa;
- Data on ROP prevalence in developing countries is scanty;
- Black race is protective to development of vision- threatening ROP.

### What this study adds

- This study contributes to data on ROP prevalence rates in Kenya and sub- Saharan Africa;
- It shows that ROP prevalence in low income countries can match that in middle and high income countries when neonatal services are well developed;
- It shows that the prevalence of vision-threatening ROP in black infants is comparable to that of infants of other races.

## Competing interests

The authors declare no competing interests.
